# Unraveling the Fungi–Cancer Connection

**DOI:** 10.34133/research.0931

**Published:** 2025-10-03

**Authors:** Weici Liu, Kai Zhu, Lu Wang, Ning-Ning Liu, Wenjun Mao

**Affiliations:** ^1^ Department of Thoracic Surgery, The Affiliated Wuxi People’s Hospital of Nanjing Medical University, Wuxi People’s Hospital, Wuxi Medical Center, Nanjing Medical University, Wuxi 214023, China.; ^2^ State Key Laboratory of Systems Medicine for Cancer, Center for Single-Cell Omics, School of Public Health, Shanghai Jiao Tong University School of Medicine, Shanghai 200025, China.

## Abstract

Accumulating evidences indicated that both the intestinal and intratumor microbiota substantially impacted the cancer initiation, progression, and therapeutic responses. Currently, the bacterial roles in cancer are particularly emphasized, while the fungal roles are just coming onto the stage. In this Perspective, we mainly focused on the fungi–tumor axis to understand the mycobiome–cancer connection, aiming to answer the questions that cause confusion and impede translational potential: (a) Why should we value the role of mycobiome in oncological research? (b) What will the relationship between fungi and bacteria be in cancer progression? (c) How will the fungi impact cancer? (d) Can we target fungi for development of intervention strategies in anticancer treatment? (e) Will the effort and investment pay back in mycobiome-driven cancer research?

## Wide Distribution of Fungi across Tumor Tissues and Implications in Cancer Biology

It is well acknowledged that there exists lower microbial biomass in tumor tissues than in gastrointestinal tract or skin. Particularly, the relative abundance of fungal biomass is even much lower than bacteria across tissues (fungi ≈ 4% to 13.3% versus bacteria ≈ 86.7% to 96%) [1,2]. Hence, advanced microbial detection methods with high depth, high coverage, and high sensitivity are required to uncover the extremely low fungal abundance (about one fungus per 10^4^ tumor cells) and isolate the live fungal species, such as internal transcribed spacer (ITS) sequencing, 18*S* ribosomal DNA (rDNA) sequencing, deep shotgun metagenomics sequencing, whole-genome sequencing, single-microbe sequencing, and single-cell host–microbiota interactome [2–5]. By virtue of these methods, tumor-specific mycobiomes in multiple tumor tissues across different body sites were discovered and the tumor-associated cell-free fungal nucleic acids with potential diagnostic or prognostic values were detected as potential biomarkers [2,3].

Aside from these fungal commonalities (including wide distribution, low abundance but high signal activity, and potential diagnostic/prognostic values) across cancer types, the fungal specificities in certain tumors are also worth noting. Previous investigations revealed several fungal genera present in the primary tumor sites, mainly including *Candida* and *Malassezia*. A pan-cancer research identified *Blastomyces* in lung cancer, *Cladosporium sphaerospermum* and *Malassezia globosa* in breast cancer, *Candida* in stomach and colon cancers, *Phaeosphaeriaceae* in ovarian cancer, and *Candida* and *Lactobacillus* in head-neck cancers [3]. Additionally, some single-cancer studies also revealed *Malassezia* in pancreatic cancer [6], *Alternaria arborescens* in non-small-cell lung cancer (NSCLC) [7], and *Aspergillus sydowii* in lung adenocarcinoma (LUAD) [8]. Furthermore, a side-by-side pan-cancer analysis, utilizing fungal histological staining on tissue microarrays, visualized the intracellular distribution of *Aspergillus* within multiple tumor tissues [2], which suggested the crucial role of fungi in regulating cancer progression. The diverse intratumor mycobiome signatures can be attributable to different cancer types and the heterogeneous tumor microenvironment (TME). It is fully recognized that one fungal species may exist in many cancer types and one cancer type may have many fungal species. However, the simple correlation does not necessarily imply that cancer-specific fungi play a part in cancer biology; alternatively, the enrichment of specific fungi could be the consequence of cancer initiation and development. It is essential to uncover the intrinsic causal relationship between specific fungal species and particular cancer types.

## The “Fungi–Bacteria–Host” Micro-Ecological Network during Cancer Progression

The microbiome, a highly dynamic and complicated ecological network, consists of bacteria, fungi, viruses, archaea, and certain parasites (e.g., protists). Although the bacterial kingdom dominates the integral microbiome, mycobiome also plays a crucial role during cancer progression. Fungi often thrive in hypoxic/acidic tumor regions (unlike most bacteria), potentially creating spatial heterogeneity in microbial interactions [9].

**Fig. 1.**
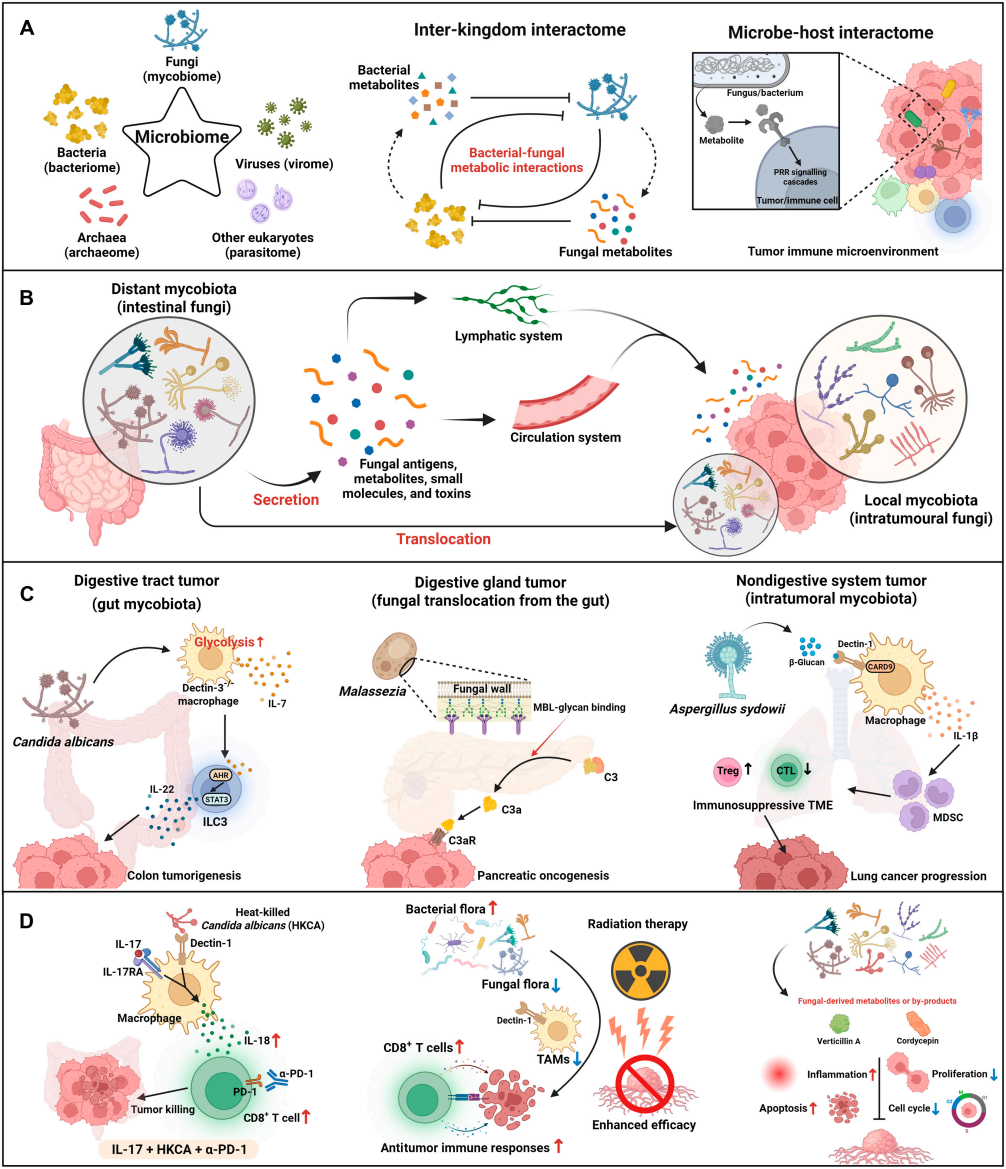
Delving into the role of mycobiome in cancer. (A) The microbiome is a dynamic and complicated collection of bacteria (bacteriome), fungi (mycobiome), viruses (virome), archaea (archaeome), and certain eukaryotes (parasitome). In the context of the microbiome, fungal and bacterial species exert negative effects on each other, and their inter-kingdom interactions are mainly dependent on microbe-derived metabolites, toxins, or small proteins. Additionally, these factors produced by microbes can also regulate the tumor apoptosis process or antitumor immunity through certain signalings (e.g., pattern recognition receptor signaling cascades), thus mediating the microbe–host interactions. (B) The distant and local mycobiota exert synergistic impacts on TME. Aside from the fungal translocation from distant tissues to local tumor tissues, a wide range of toxins, metabolites, and small molecules from non-adjacent fungi can also activate downstream cellular receptors and signalings within the TME after traveling through the circulation or lymphatic systems and then influence tumorigenesis, progression, and therapeutic response. (C) Either intestinal or intratumor fungi play cancer-promoting roles across multiple cancer types. *C. albicans* suppresses host intestinal immunity and promote colon tumorigenesis by regulating the crosstalk between *Dectin-3^−/−^* macrophages and innate lymphoid cells. In the case of pancreatic ductal adenocarcinoma, the intestinal fungi are able to induce pancreatic oncogenesis by translocation from gut to pancreas and activation of downstream complement cascades. Intratumor *A. sydowii* leads to lung cancer progression by shaping an immunosuppressive TME. (D) Fungi and mycobiota have great potential in anticancer treatment. IL-17A, in combination with heat-killed *C. albicans*, synergistically sensitizes colorectal tumors to immune checkpoint inhibitors such as α-PD-1. The interactions between bacteria and fungi in the intestine can determine the efficacy of radiation therapy by influencing antitumor immunity. Certain fungal-derived metabolites or by-products have antitumor potential by modulating the inflammatory milieu, cell cycle, cellular proliferation, and apoptosis. PRR, pattern recognition receptor; AHR, aryl hydrocarbon receptor; STAT3, signal transducer and activator of transcription 3; ILC3, group 3 innate lymphoid cells; MBL, mannose-binding lectin; CARD9, caspase recruitment domain-containing protein 9; MDSC, myeloid-derived suppressor cell; Treg, regulatory T cell; CTL, cytotoxic T lymphocyte; TME, tumor microenvironment; TAMs, tumor-associated macrophages; HKCA, heat-killed *C. albicans*.

Actually, fungi and bacteria tend to present synergistic or antagonistic relationships in tumor (Table S1). The bacteria–fungal metabolic interactions can alter the fungal and bacterial microbiome structure, indirectly influencing tumorigenesis and progression. Specifically, certain fungal and bacterial species may exert negative effects to each other, which are mediated by microbe-derived metabolites, toxins, or small proteins (Fig. 1A) [10]. For instance, bacteria-derived short-chain fatty acids (SCFAs) have been revealed as potential regulators of fungal cell biology, including inhibition of *Candida albicans* growth, reduction of yeast-to-hypha morphogenesis, and remodeling of cell wall structure [11]. In lung cancer patients, intestinal *Candida* overgrowth results from an increase in lactate-producing bacteria, coinciding with a decrease in SCFA-producing bacteria [12]. On the other hand, the *Candida* expansion in the gut can lead to marked reductions in anaerobic bacterial burden and diversity [13]. In addition, these microbe-secreted components can simultaneously impact the microbe–host interactions within the TME. Typically, fungal pathogen-associated molecular patterns (PAMPs) (e.g., β-glucans) or bacterial metabolites are capable of modulating host cellular receptors (e.g., Dectin-1 for fungi versus Toll-like receptors for bacteria) and downstream signaling cascades [10], further regulating tumor biology or antitumor immunity (Fig. 1A).

Intriguingly, commensalism of fungi and bacteria can be leveraged as microbial biomarkers for cancer risk stratification, early cancer diagnosis, and even treatment response prediction [2,14,15]. The multi-kingdom microbial biomarker that incorporates differentially expressed bacterial and fungal features surpasses the single kingdom in early screening of colorectal cancer [14] and accurately predicts immunotherapeutic responses at the pan-cancer level [15]. Thus, it is promising to move beyond the cancer mycotypes to delve into bacteria–fungi–host triple interactions in future investigations. Given the other contributors in the microbiome and TME to cancer progression, extensive explorations should be extended to mycobiome–bacteriome–archaeome–virome–immunome interactions, aiming to delineate a panorama of microbiota–host interactions during cancer development.

## Carcinogenic Effects of Fungi through Multiple Mechanisms

When mentioning the tumor-associated mycobiome, equal attention should be paid to both distant fungi (e.g., intestinal mycobiota) and local fungi (e.g., intratumor mycobiota), although it is spatially considered that local fungi play a more proximate and direct role in tumorigenesis and progression. The fungal translocation from distant niches to local tumor tissues may suggest a potential combinatorial effect of distant and intratumor mycobiota on the regulation of the TME. For example, the distant fungi-derived molecular components can enter into the blood circulation or the lymphatic system and then be transferred to the tumor tissues, further activating downstream receptors and signalings that modulate the TME (Fig. 1B) [1,16].

Recent studies have shown that the proximity of the primary tumor site to the gut is a consideration in determining whether intestinal or intratumor fungi play a more critical role in tumorigenesis and progression (Fig. 1C). In colorectal cancer, its initiation is partly attributable to the mycobiota dysbiosis in the gut, where intestinal fungi have remarkable and direct interplay with tumor cells and immune cells. For instance, *C. albicans* promotes glycolysis and interleukin-7 (IL-7) production in *Dectin-3^−/−^* macrophages, and then IL-7 binds to aryl hydrocarbon receptor (AHR) and activates signal transducer and activator of transcription 3 (STAT3) signaling to induce IL-22 secretion from group 3 innate lymphoid cells (ILC3s), thus inhibiting host immunity and driving colon tumorigenesis [17]. For digestive gland tumors, they are in close proximity to the gut, and hence, fungal translocation from the gut is probably responsible for the origin of intratumor mycobiota. As an example, *Malassezia* migrating from the intestinal lumen to the pancreas can activate the mannose-binding lectin (MBL)/C3a/C3aR axis to promote pancreatic oncogenesis [6]. For tumors in nondigestive system, intratumor mycobiota may have more direct carcinogenic influences than those non-neighboring fungi in the gut. A recent study focusing on intratumor fungi in LUAD has unraveled that tumor-resident *A. sydowii* regulates the β-glucan/Dectin-1/CARD9 axis in macrophages to promote IL-1β-mediated expansion and activation of myeloid-derived suppressor cells, thus contributing to an immunosuppressive TME for lung cancer development [8]. Additionally, certain fungal metabolites (e.g., mycotoxins) also exert carcinogenic effects, and a typical example is hepatocellular carcinoma-inducing aflatoxins that are produced by *Aspergillus*. Lately, aflatoxin B_1_ was found to synergistically disrupt the p53 pathway with hepatitis B virus (HBV), remarkably enhancing the risk of hepatocarcinogenesis [18]. This discovery substantiated the latent synergism between fungi and viruses in cancer progression. Collectively, these studies shed light on the role of fungi in tumorigenesis, either as “drivers” [6,8,17] or as “co-conspirators” [18].

Notably, existing studies failed to make the distinction between fungal spore and mycelium within tumors, despite their different distributions, functions, and clinical implications in the TME—spores primarily facilitate dissemination, whereas mycelia predominantly contribute to invasion. High-throughput ITS sequencing can merely achieve the genus/species-level identification, unable to distinguish morphology. Hence, fluorescence in situ hybridization (FISH) or scanning electron microscopy (SEM) is required to differentiate spores from mycelia. Moreover, future endeavors should scale out to determine causality at the pan-cancer level and unmask the bacteria–fungi–host interplay at the single-cell level [5].

## Therapeutic Potential of Anticancer Treatment by Modulation of Mycobiome

The intricate involvement of mycobiome in tumorigenesis and progression indicates the feasibility of manipulating certain fungi to enhance antifungal immunity in conjunction with traditional antitumor immune responses (Fig. 1D). Recently, the mycobiome has been reported to restrict invasive growth of interleukin-17 receptor A (IL-17RA)-deficient tumor cells, demonstrating that increased fungal invasion can protect against tumor progression in late-stage colorectal cancer, where IL-17RA in myeloid cells plays a pivotal role in fueling fungal-induced antitumor immunity by stimulating IL-18 secretion [19]. More translationally, the combination of recombinant IL-17 and heat-killed *C. albicans* is able to sensitize microsatellite-stable colorectal tumors to immune checkpoint blockade [19]. The gut bacteria–fungal interplay can also determine the post-radiotherapy antitumor immune responses against breast cancer and melanoma [20]. The bacterial depletion can cause overgrowth of specific fungi, which eventually decrease antitumor immunity in T cells through a dectin-1–mediated fungal sensing mechanism in pro-tumor macrophages. Therefore, it is promising to modulate the mycobiome (e.g., antimycotic-mediated fungal depletion) for effective antitumor immunity following radiation [20].

Moreover, fungal-derived metabolites or by-products have been documented to possess antitumor properties [21,22]. As a typical fungal metabolite, verticillin A can selectively inhibit histone methyltransferase to regulate the cell cycle, cellular apoptosis, stress response, and PD-L1 expression, thereby displaying potent tumor-suppressive potential [21]. Similarly, as the primary compound of *Cordyceps sinensis*, cordycepin, also termed 3-deoxyadenosine, is capable of inhibiting tumor progression, which depends on its pro-inflammatory, pro-apoptotic, and anti-proliferative mechanisms by modulating caspase, mitogen-activated protein kinase (MAPK), or glycogen synthase kinase-3β (GSK-3β) pathways [22]. Nonetheless, current evidences are mostly established on the mouse models, necessitating further translation from preclinical models to clinical tumor patients.

Fortunately, some clinical trials have preliminarily proven the clinical translation potential of antifungal drugs in tumor treatment, such as (a) itraconazole for esophageal cancer (NCT02749513) and lung cancer (NCT02157883), (b) ketoconazole for breast cancer (NCT00544804) and prostate cancer (NCT00460031), and (c) 5-fluorocytosine for glioma (NCT01470794 and NCT02414165). Nonetheless, antifungals face greater host toxicity risks due to eukaryotic cell similarity than antivirals and antibiotics, necessitating further evaluation of their safety and toxicity. Moreover, drug resistance risks and individual differences may also pose latent concerns. In short, it is promising for next-generation anticancer treatments to be optimized not only by regulating the intestinal/intratumor microbiome (e.g., by antibiotics, antifungals, or even microbiota transplant) but also by leveraging bacterial or fungal metabolites as adjuvants.

Particularly, as for the fecal mycobiota transplantation, there is quite a long road for its feasibility in cancer treatment. For one thing, transplantation interventions targeting the fungal microbiota are still in the exploratory stage, lacking systematic clinical trial data to support their success in replicating fecal microbiota transplantation (FMT). For another, the intestinal microbiota is a micro-ecological community and the mycobiota accounts for a low proportion. Therefore, extracting fungi from the healthy donor’s fecal microbiota is difficult and may compromise flora integrity. Simultaneously, it is challenging to solve the issues of standardized preparation, safety evaluation, and individualized treatment. These hurdles in front of mycobiome modulation remain to be addressed.

## Stop Neglecting the Mycobiome-Driven Cancer Research and Time Will Tell

**Fig. 2.**
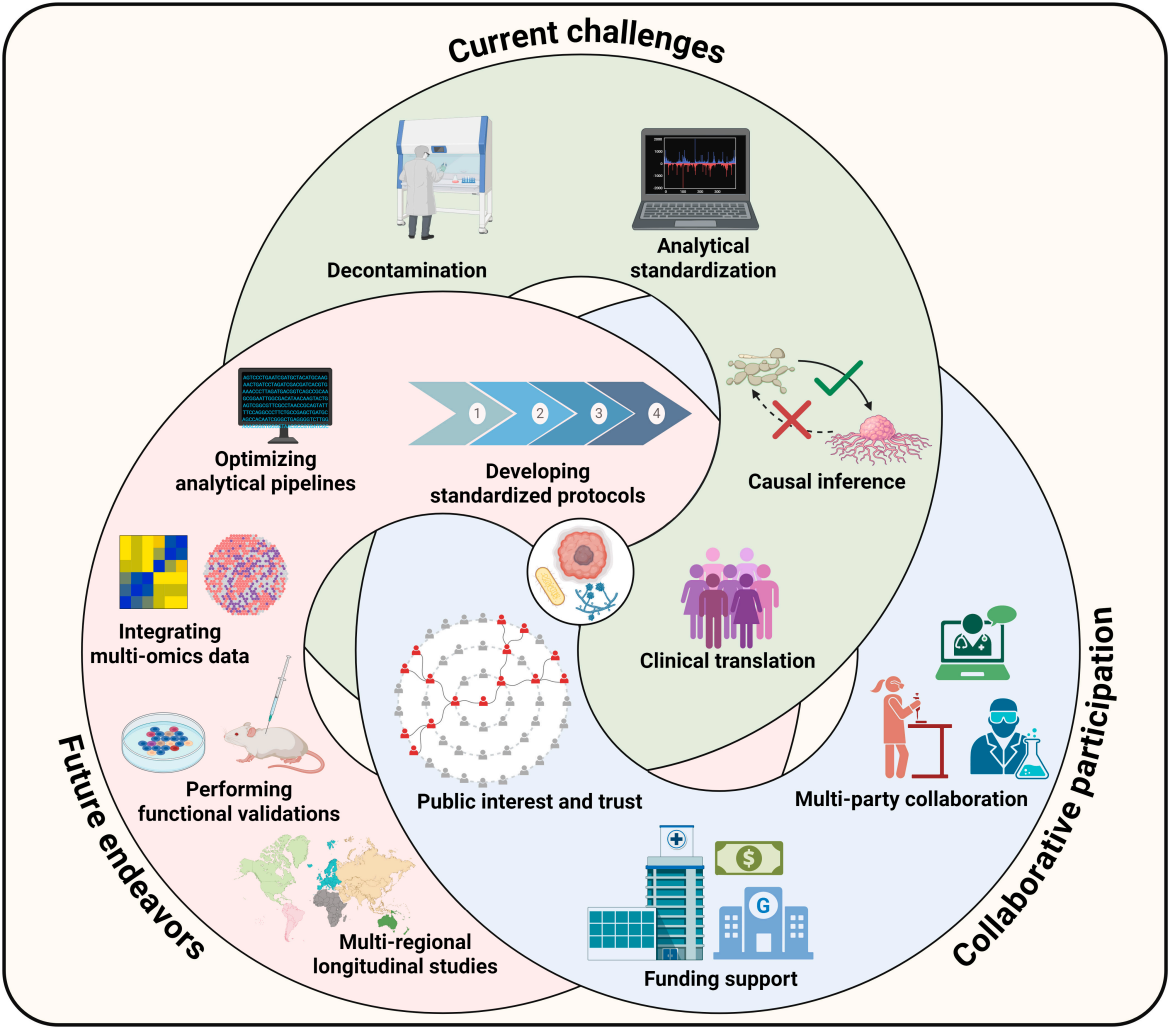
The long way ahead for mycobiome-driven cancer research.

Currently, the uneven research landscape of cancer microbiome features a focus on bacteriome in tumorigenesis and progression, with mycobiome lagging far behind [9,23]. However, fungal flora has equally crucial roles and great potential in the diagnosis and treatment of cancer, such as multiple fungal combinations or bacteria–fungal combinations as latent biomarkers for malignancy, as well as mycobiota modulation or fungal-targeted interventions in synergy with existing antitumor therapies. We herein provide answers to just a few representative questions in the underexplored field of cancer mycobiome, in the hope of progressively establishing public interest and trust to advocate for more profound insights into the fungal role in cancer. Although embracing great promise, studies on cancer mycobiome are still confronted with a range of challenges from decontamination and analytical standardization to causal inference and clinical translation [23]. Consequently, next endeavors should include utilizing fungal-enriched sequencing methods, developing standardized protocols, adopting optimal analytical pipelines, integrating multi-omics data, performing functional validations, and conducting multi-regional, multi-cohort longitudinal studies. There is still a long way ahead to secure a bright future for mycobiome-driven cancer research and ultimately benefit cancer patients, and it will require public confidence in the cancer mycobiome, financial support funded by the government, and the collective collaboration of microbiologists, computational biologists, and clinical oncologists (Fig. 2).
