# Supplementary material for: Unraveling the Fungi–Cancer Connection
Source: Research (Wash D C). 2025 Oct 3;8:0931. doi: 10.34133/research.0931 (PMC12491779; doi:10.34133/research.0931)
Supplement: Supplementary 1 — Table S1 [file research.0931.f1.docx]

**Unraveling the Fungi-cancer connection**

Weici Liu, Kai Zhu, Lu Wang, Ning-Ning Liu, Wenjun Mao

**Correspondence to**:

maowenjun1@njmu.edu.cn (W.M.); liuningning@shsmu.edu.cn (N.L.)

**Lead contact**:

Wenjun Mao, M.D, Professor.

Department of Thoracic Surgery

The Affiliated Wuxi People’s Hospital of Nanjing Medical University

No. 299 Qingyang Rd., Wuxi, 214023, China

1. mail: maowenjun1@njmu.edu.cn

**Supplementary materials**

**Table S1**. The synergistic and antagonistic relationships between fungi and bacteria in tumor progression

**Table S1**. The synergistic and antagonistic relationships between fungi and bacteria in tumor progression

| Relationship | Evidence source | Fungal-bacterial combination | Tumor type | Key results or mechanisms | Ref |
| --- | --- | --- | --- | --- | --- |
| Synergy/  antagonism | 16S rRNA gene sequencing + ITS-1 gene sequencing + PCR + patient fecal samples | 2 bacteria (*Fusobacterium nucleatum*, *Bifidobacterium bifidum*) + 2 fungi (*Candida albicans*, *Saccharomyces cerevisiae*) | Colorectal cancer | High abundance of *Fusobacterium nucleatum*, *Candida albicans*, and *Saccharomyces cerevisiae*, but low abundance of *Bifidobacterium bifidum* | [1] |
| Synergy/  antagonism | ITS-2 gene sequencing + shotgun metagenomics sequencing | 2 bacteria (lactate-producing bacteria, SCFAs-producing  bacteria) + *Candida* | Lung cancer | Intestinal *Candida* overgrowth results from an increase in lactate-producing bacteria, coinciding with a decrease in SCFAs-producing bacteria. | [2] |
| Synergy | 16S rRNA gene sequencing + ITS-2 gene sequencing + AOM/DSS-induced CRC mouse models | *Lactobacillus kefiranofaciens* JKSP109 + *Saccharomyces cerevisiae* JKSP39 | Colorectal cancer | Fungal-bacterial co-culture alleviates inflammation and colorectal carcinogenesis in mice, with high content of short chain fatty acids in fecal samples. | [3] |
| Synergy | Flow-cell biofilm formation + FISH staining | 2 bacteria (*Actinomyces naeslundii,* *Streptococcus mutans*) + 1 fungi (*Candida albicans)* | Oral cancer | *Candida albicans*, *Actinomyces naeslundii* and *Streptococcus mutans* form polymicrobial biofilms to modulate cancer progression by regulating extracellular matrix adhesion, epithelial-mesenchymal transition, and pro-inflammatory cytokines expression. | [4] |
| Antagonism | 16S rRNA gene sequencing + ITS-1 gene sequencing | Bacterial microbiota + 2 fungi (*Saccharomyces*, *Candida*) | Breast cancer;  Melanoma | Antibiotic-mediated depletion of bacteria leads to overgrowth of commensal fungi (mainly *Saccharomyces* and *Candida*), orchestrating the immunosuppressive TME and reducing responsiveness to radiation. | [5] |

Abbreviations: ITS, internal transcribed spacer; PCR, polymerase chain reaction; SCFAs, short chain fatty acids; AOM/DSS, azoxymethane/dextran sulfate sodium; CRC, colorectal cancer; FISH, fluorescent in situ hybridization; TME, tumor microenvironment.

**References**:

1. Li X, Feng J, Wang Z, Liu G, Wang F (2023) Features of combined gut bacteria and fungi from a Chinese cohort of colorectal cancer, colorectal adenoma, and post-operative patients. Front Microbiol 14:1236583

2. Seelbinder B, Lohinai Z, Vazquez-Uribe R, et al (2023) Candida expansion in the gut of lung cancer patients associates with an ecological signature that supports growth under dysbiotic conditions. Nat Commun 14:2673

3. Zeng X, Jia H, Shi Y, Chen K, Wang Z, Gao Z, Yuan Y, Yue T (2022) Lactobacillus kefiranofaciens JKSP109 and Saccharomyces cerevisiae JKSP39 isolated from Tibetan kefir grain co-alleviated AOM/DSS induced inflammation and colorectal carcinogenesis. Food Funct 13:6947–6961

4. Arzmi MH, Cirillo N, Lenzo JC, Catmull DV, O’Brien-Simpson N, Reynolds EC, Dashper S, McCullough M (2019) Monospecies and polymicrobial biofilms differentially regulate the phenotype of genotype-specific oral cancer cells. Carcinogenesis 40:184–193

5. Shiao SL, Kershaw KM, Limon JJ, et al (2021) Commensal bacteria and fungi differentially regulate tumor responses to radiation therapy. Cancer Cell 39:1202-1213.e6
